# Suppression of estrogen receptor beta classical genomic activity enhances systemic and adipose-specific response to chronic beta-3 adrenergic receptor (β3AR) stimulation

**DOI:** 10.3389/fphys.2022.920675

**Published:** 2022-09-16

**Authors:** Eric D. Queathem, Maggie Fitzgerald, Rebecca Welly, Candace C. Rowles, Kylie Schaller, Shahad Bukhary, Christopher P. Baines, R. Scott Rector, Jaume Padilla, Camila Manrique-Acevedo, Dennis B. Lubahn, Victoria J. Vieira-Potter

**Affiliations:** ^1^ Department of Nutrition and Exercise Physiology, University of Missouri, Columbia, MO, United States; ^2^ Department of Biochemistry, University of Missouri, Columbia, MO, United States; ^3^ Dalton Cardiovascular Research Center, University of Missouri, Columbia, MO, United States; ^4^ Department of Biomedical Sciences, University of Missouri, Columbia, MO, United States; ^5^ Internal Medicine-Division of Gastroenterology and Hepatology, University of Missouri, Columbia, MO, United States; ^6^ Research Service, Truman VA Memorial Hospital, Columbia, MO, United States; ^7^ Division of Endocrinology, Diabetes and Metabolism, Department of Medicine, University of Missouri Columbia School of Medicine, Columbia, MO, United States

**Keywords:** adipocyte browning, insulin resistance, adipocyte mitochondria, energy expenditure, estrogen signaling

## Abstract

White adipose tissue (WAT) dysfunction independently predicts cardiometabolic disease, yet there is a lack of effective adipocyte-targeting therapeutics. B3AR agonists enhance adipocyte mitochondrial function and hold potential in this regard. Based on enhanced sensitivity to B3AR-mediated browning in estrogen receptor (ER)alpha-null mice, we hypothesized that ERβ may enhance the WAT response to the B3AR ligand, CL316,243 (CL).

**Methods:** Male and female wild-type (WT) and ERβ DNA binding domain knock-out (ERβ_DBD_KO) mice fed high-fat diet (HFD) to induce obesity were administered CL (1 mg/kg) daily for 2 weeks. Systemic physiological assessments of body composition (*EchoMRI*), bioenergetics (*metabolic chambers*), adipocyte mitochondrial respiration (*oroboros*) and glucose tolerance were performed, alongside perigonadal (PGAT), subcutaneous (SQAT) and brown adipose tissue (BAT) protein expression assessment (*Western blot*). Mechanisms were tested *in vitro* using primary adipocytes isolated from WT mice, and from Esr2-floxed mice in which ERβ was knocked down. Statistical analyses were performed using 2 × 2 analysis of variance (ANOVA) for main effects of genotype (G) and treatment (T), as well as GxT interactions; t-tests were used to determine differences between *in vitro* treatment conditions (SPSS V24).

**Results:** There were no genotype differences in HFD-induced obesity or systemic rescue effects of CL, yet ERβ_DBD_KO females were more sensitive to CL-induced increases in energy expenditure and WAT UCP1 induction (GxT, *p* < 0.05), which coincided with greater WAT B3AR protein content among the KO (G, *p* < 0.05). Among males, who were more insulin resistant to begin with (no genotype differences before treatment), tended to be more sensitive to CL-mediated reduction in insulin resistance. With sexes combined, basal WAT mitochondrial respiration trended toward being lower in the ERβ_DBD_KO mice, but this was completely rescued by CL (*p* < 0.05). Confirming prior work, CL increased adipose tissue ERβ protein (T, *p* < 0.05, *all*)*,* an effect that was enhanced in WAT and BAT the female KO (GxT, *p* < 0.01). *In vitro* experiments indicated that an inhibitor of ERβ genomic function (PHTPP) synergized with CL to further increase UCP1 mRNA (*p* = 0.043), whereas full ERβ protein was required for UCP1 expression (*p* = 0.042).

**Conclusion:** Full ERβ activity appears requisite and stimulatory for UCP1 expression via a mechanism involving non-classical ERβ signaling. This novel discovery about the role of ERβ in adipocyte metabolism may have important clinical applications.

## Introduction

Obesity and its associated diseases are a global epidemic (Saklayen, 2018), yet there remains a lack of effective preventative and therapeutic strategies. Given the established link between adipose tissue dysfunction and multiple metabolic diseases (Klöting et al., 2010; Haas et al., 2012; Lomonaco et al., 2012; Klöting and Blüher, 2014; Gastaldelli et al., 2017; Norheim et al., 2019; Sergi et al., 2019), therapeutics specifically targeting adipocytes are urgently needed. In this regard, from a molecular perspective, adipocyte tissue mitochondrial dysfunction plays an important role in the etiology of its dysfunction, which contributes to insulin resistance, dyslipidemia, inflammation, and ultimately, metabolic disease. Unfortunately, there are currently no effective therapeutics to selectively target adipocyte mitochondrial function. The present work may open up new therapeutic avenues in this regard.

Chronic activation of the beta-3 adrenergic receptor (β3AR) via the selective chemical ligand CL316,243 (CL) (Bloom et al., 1992) is a well-documented mechanism to enhance adipocyte mitochondrial function. CL promotes “browning” of white adipose tissue (WAT) (Ghorbani and Himms-Hagen, 1997; Kaisanlahti and Glumoff, 2019) by increasing uncoupling protein 1 (UCP1) and mitochondrial protein content, and decreasing WAT mass (Himms-Hagen et al., 1994; Yoshida et al., 1994). These effects are similar to the effects of exercise on WAT browning (De Matteis et al., 2013; Aldiss et al., 2018); therefore, CL may be considered an adipocyte-specific “exercise mimetic”. In addition to its anti-obesity effects, CL administration has also been shown to regulate blood glucose (Ghorbani and Himms-Hagen, 1997; MacPherson et al., 2014). Recent studies have shown that combining CL with antidiabetic drugs (e.g., liraglutide) (Decara et al., 2018) has additional benefits. While β3ARs are expressed in several tissues (e.g., WAT and brown adipose tissue (BAT) (Evans et al., 1996; Monjo et al., 2005; Baskin et al., 2018; Tournissac et al., 2020), kidney (Procino et al., 2016), bladder (Procino et al., 2016), colon (Evans et al., 1996; Chamberlain et al., 1999), stomach (Evans et al., 1996), prostate (Chamberlain et al., 1999), gallbladder (Baskin et al., 2018), skeletal myocytes (Chamberlain et al., 1999), and cardiomyocytes (Chamberlain et al., 1999; Balligand, 2017; Machuki et al., 2018)), they have a uniquely high level of expression on adipocytes (Evans et al., 1996; Chamberlain et al., 1999). Therefore, the beneficial effects of CL have largely been attributed to its adipocyte-specific effects. While it has been shown that CL is capable of increasing energy expenditure and promoting mitochondrial biogenesis independent of UCP1 (Granneman et al., 2003), evidence from our lab and others have indicated that UCP1 is necessary for optimal adipocyte mitochondrial function, and is protective metabolically in both sexes (Inokuma et al., 2006; Winn et al., 2017a; Winn et al., 2017b; Clookey et al., 2018). Further, given that lack of UCP1 prevents the reduction in body mass observed with chronic CL treatment (Inokuma et al., 2006), the importance of UCP1 in mediating at least some of the beneficial effects of CL, namely its energy expenditure increasing effect, must be recognized. However, the mechanism by which CL increases UCP1 remains largely known*.*

Estrogen (E2) is classically thought of as the female sex hormone, but has plays important roles in both female and male physiology (Cooke et al., 2017). Further, E2 regulates mitochondrial biogenesis (Klinge, 2008; Chen et al., 2009; Klinge, 2017) and reactive oxygen species (ROS) production (Burris and Krishnan, 2005; Razmara et al., 2007; Razmara et al., 2008; Liao et al., 2019) in a variety of cell types. We have been studying its adipocyte-specific actions for over a decade. WAT is heavily influenced by E2 (Gray et al., 1981; Pedersen et al., 1992), and is the major site of E2 synthesis in males and post-menopausal women (Simpson et al., 1994; Simpson, 2003; Barakat et al., 2016). It expresses both E2 receptor alpha (ERα) and ERβ (Pedersen et al., 1992; Crandall et al., 1998), the two most well-studied ERs. Both ERα and ERβ share the conserved steroid nuclear transcription factor structure. Further, both ERα and ERβ influence mitochondrial function (Monje and Boland, 2001; Klinge, 2008; Jia et al., 2014; Klinge, 2017). ERα was recently shown to be necessary for optimal adipocyte mitochondrial remodeling and uncoupling activity (Zhou et al., 2020). Unfortunately, targeting nuclear activities of ERα is not recommended because of the feminizing and cancer-promoting effects of ERα signaling. In fact, E2 signaling pathways have historically been underutilized to treat metabolic diseases due to the feminizing and cancer promoting effects of E2, but these effects are now known to be attributed to ERα (Clusan et al., 2021; Uenoyama et al., 2021). Unlike ERα, which is expressed at much greater levels in female WAT, ERβ is expressed at the same level in males and females (Porter et al., 2020; Queathem et al., 2021) and does not have feminizing effects (Yepuru et al., 2010; González-Granillo et al., 2019a; González-Granillo et al., 2019b). Therefore, ERβ is emerging as an important potential molecular target to treat metabolic dysfunction in both sexes. Notably, many of the beneficial mechanisms of ERβ in other cell types have been attributed to improvements in mitochondrial function (Liao et al., 2015), and ERβ has indeed been found in the mitochondria of multiple cell types (Chen et al., 2004; Yager and Chen, 2007; Simpkins et al., 2008; Burstein et al., 2018; Liao et al., 2019). The potential physiological relevance of its mitochondrial localization is exemplified by the following examples: ERβ is required for exercise-induced myocardial hypertrophy and mitochondrial remodeling (Dworatzek et al., 2014); is involved in regulating permeability transition in brain mitochondria (Burstein et al., 2018); and protects against endometriosis pathogenesis via modulating mitochondrial function (Liao et al., 2019). We hypothesize that, in adipose tissue, ERβ plays a critical and necessary role in mitochondrial metabolism via its relationship with UCP1. Given that UCP1 buffers mitochondrial ROS (Shabalina et al., 2006; Oelkrug et al., 2014; Chouchani et al., 2016; Jia et al., 2019), and the important yet elusive role of E2 in regulating ROS, the role of ERβ in regulating ROS, UCP1, and mitochondrial function in WAT requires more research attention.

ERβ was first discovered in 1996 in rat prostate and ovary (Kuiper et al., 1996) and was subsequently identified in humans (Mosselman et al., 1996). The first animal model designed to lack ERβ activity was generated in the lab of Oliver Smithies (Krege et al., 1998) by disrupting exon 3 of the DNA binding domain (DBD). Because that model lacked nuclear DNA binding of ERβ, and thus was deficient in classical genomic activities, the model was considered an ERβ knock-out mouse, even though it still expressed a mutant ERβ protein lacking the DBD. Later studies using alternative means of disrupting ERβ gene function revealed different phenotypes to this original model (Dupont et al., 2000; Shughrue et al., 2002; Antal et al., 2008). Earlier studies demonstrated that ERβ is a weaker activator of estrogen response elements (EREs), compared to ERα (Kuiper et al., 1997; McInerney et al., 1998), which suggested that ERβ may function via ERE-independent (i.e., non-classical genomic) mechanisms. Subsequent studies supported that some outcomes of ERβ activation involve non-ERE binding events, mediated through ERβ-protein interactions (O’Lone et al., 2004). Moreover, membrane-bound ERβ may also indirectly affect nuclear gene expression by triggering intracellular signaling cascades culminating in changes in DNA transcription and translation. Given this complexity, it is important to begin to interrogate unique functions of ERβ. Specific genetic manipulations that silence only certain functional aspects of this receptor offer us a way to do this. While much remains unknown about novel non-classical genomic functions of ERβ, our present work supports the exciting possibility that these functions may be targeted to enhance adipocyte mitochondria and mitigate obesity-related disease. This may be surprising to readers who are aware of the early studies that hypothesized ERβ functioned in *opposition* to ERα in WAT (Naaz et al., 2002); however, the role of non-classical genomic ERβ signaling pathways were not considered in those studies. Since then, there is an emerging understanding of the importance of non-classical genomic signaling through ERβ. For example, ERβ is known to negatively regulate peroxisome proliferating receptor gamma (PPARγ) by competing for a shared coactivator (Foryst-Ludwig et al., 2008) resulting in suppression of adipocyte proliferation (i.e., an anti-obesity effect). This is just one illustration of ERβ′s DBD-independent (i.e., non-classical) functions.

Our research focuses on the mechanistic role of ERβ in mediating protective metabolic effects in adipocyte mitochondria in the setting of obesity (Clookey et al., 2019; Winn et al., 2019; Porter et al., 2020; Zidon et al., 2020). Collectively this work has shown that: 1) absence of ERβ, *but not ERα,* exacerbates ovariectomy-induced WAT dysfunction in mice (Zidon et al., 2020); 2) CL completely rescues HFD-induced adipocyte and systemic metabolic dysfunction in both sexes while also increasing ERβ expression specifically in WAT (Clookey et al., 2019; Queathem et al., 2021); 3) ERα is not required for the CL-induced browning of WAT and, contrarily, ERα-null mice (with intact signaling through ERβ) are more sensitive to CL (Clookey et al., 2019). These findings led to the present investigation of the role of ERβ non-classical genomic (i.e., DBD-independent) functions in CL-induced WAT browning.

We hypothesize that the beneficial effects of CL on WAT and systemic metabolism will be enhanced in an ERβ-DBD-knock-out model (ERβ_DBD_KO) due to an increase in non-classical genomic (e.g., mitochondrial) ERβ activity. This model lacks the ability to classically activate an ERE, yet still retains non-classical genomic (i.e., ERE- and DBD-independent) functions of ERβ. We treated male and female wild-type and ERβ_DBD_KO mice with CL daily for 2-weeks and then used a systems physiology approach to assess the effect of CL on energy balance, fuel oxidation, glucose tolerance, body composition, WAT and BAT metabolism, and *ex vivo* mitochondrial oxygen consumption. To determine the adipocyte-specific mechanism driving the protective metabolic effects of CL, we measured an array of proteins involved in mitochondrial metabolism, E2 signaling and adipocyte health. Additional *in vitro* experiments were conducted using primary adipocytes harvested from 1) wild-type mice treated with CL *in vitro* with and without a ligand known to suppress classical genomic ERβ activity (PHTPP) (Compton et al., 2004; Hsu et al., 2014), and 2) from an Ers2-lox P floxed mouse, treated with CRE-adenovirus *in vitro* to suppress ERβ expression*.* Collectively, our *in vivo* and *in vitro* results indicate that the adipocyte-specific increase in energy expenditure and UCP1 in response to β3AR activation is enhanced via suppression of classical genomic ERβ signaling. However, complete suppression of ERβ protein in adipocytes abolishes UCP1 expression. Thus, not only did we discover that the DBD of ERβ is *not required* for CL-induced WAT browning, our data support that isolation and/or activation of *non-classical ERβ activity* (hypothetically, mitochondrial recruitment and localization) may enhance CL’s adipocyte-specific metabolic effects. To summarize, the presence of ERβ is requisite for adipocyte UCP1 expression, but the mechanism involves a function of ERβ that is independent of its DBD.

## Materials and methods

### Animal model and experimental design

**FIGURE 1:**
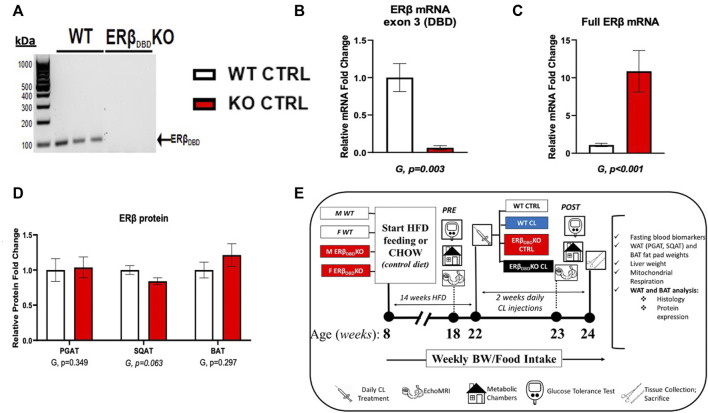
Model validation and study design. Estrogen receptor beta DNA binding domain knock-out (ERβ_DBD_KO) mice express ERβ, but lack exon3, the gene encoding the DBD. **(A)** Genotype validation via PCR. Forward primer (5′-3′): TGT​TAC​GTA​GTC​CAA​GCG​CCA​A; reverse primer (5′-3′): CAT​CCT​TCA​CAG​GAC​CAG​ACA. **(B)** Confirmation of lack of Esr2 DBD mRNA measured via qRT-PCR. Forward primer (5′-3′): TGT​TAC​TAG​TCC​AAG​CGC​CAA; reverse primer: CAT​CCT​TCA​CAG​GAC​CAG​ACA; ncbi ref seq: NM_010157.3. **(C)** Confirmation of Esr2 mRNA expression in ERβ_DBD_KO mouse. Forward primer (5′-3′): CTC​AAC​TCC​AGT​ATG​TAC​CC; reverse primer (5′-3′): CAT​GAG​AAA​GAA​GCA​TCA​GG; ncbi ref seq: NM_010157.3. **(D)** ERβ protein expression measured via western blot in perigonadal (PGAT), subcutaneous (SQAT), and brown (BAT) adipose tissue. Representative blots can be found in Supplementary Figure S1. **(E)** Overview of study design. All data are presented as mean ± SEM. *N* = 14–32/group. Statistical differences were assessed via *t*-test and presented below graphs. *p*-values < 0.05 were accepted as statistically significant.

Heterozygote ERβ_DBD_−/+ mice on a C57/BL6J background were bred at our facility to produce homozygote (ERβ_DBD_−/−) (i.e., ERβ_DBD_KO) and littermate wild-type (i.e., WT) male and female mice (both sexes were used), as previously described (Krege et al., 1998; Clart et al., 2021). The WT mice used here as controls were also used in a previous publication, where we investigated sex differences in CL-mediated browning (Queathem). Briefly, development of the ERβ_DBD_KO mouse was accomplished by homologous recombination and insertion of a neomycin sequence containing premature stop codons and polyadenylation sequences into a Not1 site in exon 3 of the mouse ERβ gene (Esr2), encoding a zinc finger of the DBD. Figure 1 providesmodel confirmation via positive PCR genotyping using tail snips (Figure 1A), confirmation of lack of exon 3 of Esr2 via qRT-PCR (Figure 1B), confirmation of the presence of a mutated form of the Esr2 gene via qRT-PCR (Figure 1C), and confirmation of the presence of a mutated ERβ protein via Western blot (Figure 1D). Collectively this shows that ERβ_DBD_KO mice lack exon 3 encoding the DBD of ERβ (G, *p* = 0.003), but retain normal levels of ERβ protein across fat depots. Mice were fed a high sucrose, high-fat-diet (HFD) consisting of 46.4% kcal from fat, 36% carbohydrate, and 17.6% protein, with a density of 4.68 kcal per Gram (Test Diet, St. Louis MO, #1814692). Separate groups of mice were fed a normal chow (CHOW) diet consisting of 13% kcal from fat, 57% kcal from carbohydrate, and 30% kcal from protein, with a density of 3.3 kcal per Gram (LabDiet, St. Louis, MO, United States, #5001) as a comparison to confirm HFD-induced obesity in experimental mice. Animals were pair-housed (within group and same sexes housed together) at 28°C (i.e., thermoneutral conditions), in a light cycle from 0,700 to 1900. Prior to and following drug treatments, the mice were assessed for body composition and glucose tolerance. Subsequently, the HFD-fed obese animals began a 14-days regiment of daily intraperitoneal injections of CL316,243 (#C5976, Sigma-Aldrich, dose: 1 μg/g body weight) (CL) or equal volume of saline vehicle (CTRL). Injections occurred at 08:00 each morning during the treatment period. The HFD-fed treatment groups (i.e., WT-CTRL, WT-CL, KO-CTRL, KO-CL; *n* = 7–16/group) were euthanized at ∼24 weeks of age following a 5-h fast. Blood and tissues (PGAT, SQAT, BAT and liver) were collected, weighed, and either snap-frozen in liquid nitrogen and stored at −80°C until analysis or, for histology, fixed in 10% formalin. Figure 1E depicts the study timeline and design.

### Body composition and tissue weights

Body composition, including % lean and % fat mass, was measured by a nuclear magnetic resonance imaging whole-body composition analyzer (EchoMRI 4 in 1/1,100; Echo Medical Systems, Houston, TX, United States). Upon sacrifice, visceral (perigonadal) WAT (PGAT), subcutaneous (inguinal) WAT (SQAT), interscapular brown adipose tissue (BAT), and liver tissues were weighed to assess body composition and fat distribution.

### Histological analysis

Formalin-fixed samples were processed through paraffin embedment, sectioned at 5 µm (visceral (PGAT), subcutaneous (SQAT) WAT, and interscapular BAT) and stained with H&E. Sections were evaluated via an Olympus BX34 photomicroscope (Olympus, Melville, NY, United States) and images were taken via an Olympus SC30 Optical Microscope Accessory CMOS color camera. Adipocyte size was calculated from three independent regions of the same ×40 objective fields for SQAT, PGAT, and interscapular BAT depots (∼75 adipocytes/animal). Cross-sectional areas of the adipocytes were obtained from perimeter tracings using ImageJ software, as previously described (Wainright et al., 2015). An investigator blinded to the groups performed all procedures. Note that the representative images from the WT control mice were previously published in the investigation of sex differences in CL-mediated browning (Queathem).

### Energy intake and expenditure assessment

Indirect calorimetry was utilized before and during CL treatment (i.e., 1 week following the start of treatment). Briefly, animals were placed in indirect calorimetry chambers (Promethion; Sable Systems International, Las Vegas, NV, United States) to assess metabolic activity parameters, including total energy expenditure (TEE), spontaneous physical activity (SPA), and respiratory quotient (RQ). For TEE, body weight was used as a covariate. SPA was measured as the summation of x-, y-, and *z*-axis beam breaks. Each run captured at least two light and two dark cycles of each variable. Body weight and food intake were recorded on a weekly basis throughout the study.

### Glucose tolerance and fasting blood metabolic assessment

Glucose tolerance tests were performed twice on animals; once after ∼10 weeks of HFD and once after 1 week of CL treatment, ∼1 week before sacrifice. After a 5 h fast, blood glucose was measured from the tail vein (i.e., time 0) using a glucometer (Alpha Trak, Abbott Labs, Chicago, IL, United States). Glucose was administered (dose: 2 g/kg body weight) via intraperitoneal injection. Glucose measures were then taken 15, 30, 45, 60 and 120 min after the glucose bolus was administered. The glucose area under curve (AUC) from the baseline was calculated. At sacrifice, plasma was collected and plasma insulin, glucose, and non-esterified fatty acids (NEFA) were quantified by a commercial laboratory (Comparative Clinical Pathology Services, Columbia, MO, United States) using an Olympus AU680 automated chemistry analyzer (Beckman–Coulter, Brea, CA, United States) as per the manufacturer’s guidelines. The homeostasis model assessment of insulin resistance (HOMA-IR) was used as a surrogate measure of systemic insulin resistance ((fasting insulin (μU/L) × fasting glucose (mg/dl)/405.1) (Matthews et al., 1985). Adipose tissue insulin resistance (ADIPO-IR), another surrogate measure, was calculated as the product of fasting insulin (μU/L) and fasting NEFAs (mmol/L) (Lomonaco et al., 2012).

### *Ex vivo* adipocyte mitochondrial respiration

Mature adipocyte respiration was assessed using high-resolution respirometry (Oroboros Oxygraph-2k; Oroboros Instruments, Innsbruck, Austria). Briefly, isolated mature adipocytes were loaded into respiration chambers containing buffer MiR05 (100 mM sucrose, 60 mMK-lactobionate, 0.5 mM EGTA, 3 mM MgCl2, 20 mM taurine, 10 mM KH2PO4, 20 mM HEPES, adjusted to pH 7.1 with KOH at 37°C; and 1 g/L fatty acid-free BSA) to assess basal respiration. Digitonin (2 uM) was added to the chambers to permeabilize the adipocytes. Glutamate (5 mM) and malate (2 mM) were added to the chambers to assess State 2 respiration in the absence of ADP. State 3, complex I, respiration was then assessed by titration of ADP (50–200 uM). The addition of succinate (7.5 mM) allowed for the measurement of State 3, complex I and complex II respiration. Finally, maximal uncoupled respiration was assessed by the addition of carbonyl cyanide 4-(trifluoromethoxy) phenylhydrazone (0.25–0.5 uM).

### Protein isolation and western blotting

Protein was isolated from PGAT, SQAT and BAT and quantified using BCA method, then subjected to western blotting as previously described (Winn et al., 2017a). Briefly, protein samples (10 µg/lane) were separated on a 4–20% Criterion TGX SDS-PAGE gel, transferred to polyvinylidene difluoride membranes using BioRad Trans-Turbo Blot system, blocked with non-fat dry milk, and incubated with appropriate primary antibodies overnight (Supplementary Table S1 lists specific antibodies used). All three depots (PGAT, SQAT and BAT) were run individually. Blots were incubated in an appropriate secondary antibody conjugated to horseradish peroxidase, developed using Thermo Scientific SuperSignal West Femto Substrate, and images were captured using a ChemiDoc Imaging System (BioRad, Hercules, CA, United States). The intensities of individual protein bands were quantified using ImageLab (BioRad), expressed as a ratio to beta actin, and normalized to the WT-CTRL group, which was set at 1. Representative western blot images are presented in Supplementary Figure S1. AMPK activity was assessed via protein content analysis of its total and phosphorylated forms. AMPK phosphorylation on the Thr(172) residue was measured as an indication of AMPK activation (Lim et al., 2012), whereas phosphorylation on the Ser(485/491) residue was taken as indicative of AMPK inhibition (Coughlan et al., 2016).

### *In vitro* pharmacologic and adenovirus-mediated ERβ manipulations

PGAT and SQAT was harvested from male C57BL6J mice, digested in collagenase to isolate stromal vascular cells then cultured in Dulbecco’s modified eagle medium/nutrient mixture F12 media supplemented with 10% fetal bovine serum, 1% GlutaMax, 0.1% gentamicin and 0.05% insulin at 37°C and 5% CO_2_. Adipocyte differentiation was induced by supplementing media with 0.5 mM 3-isobutyl-1-methylxanithin, 1 uM dexamethasone, and 1 mM rosiglitazone for 48 h 14 days post induction, primary adipocytes were treated with either 1 uM CL, 1 uM PHTPP or a combination of both ligands. After 24 h treatment, total RNA was isolated using Qiagen’s RNeasy kit and assayed using a Nanodrop spectrophotometer (Thermo Scientific, Wilmington, DE, United States) to assess purity and concentration. First-strand cDNA was synthesized from total RNA using the High-Capacity cDNA Reverse Transcription kit (Applied Biosystems, Carlsbad, CA, United States), then quantitative real-time PCR was performed using the ABI StepOne Plus sequence detection system (Applied Biosystems). Ucp1 mRNA levels were then measured, using beta-actin as a housekeeping gene, as cycle thresholds (CT) was not different between groups. Ucp1 mRNA expression is expressed as 2^−ΔΔCT^ where ΔΔCT = Housekeeping gene CT—Ucp1 CT and presented as fold-difference compared to the control group. In separate experiments, PGAT and SQAT was harvested from one male Esr2-loxP floxed mouse cre^+/-^ and digested in 1 mg/ml collagenase. Stromal vascular cells were then grown and differentiated as above. Primary adipocytes were transfected 14 days after differentiation with 1.0 × 10^8^ plaque-forming units/ml media recombinant adenovirus (Vector Biolabs, Malvern, PA) containing Cre recombinase (CRE), or eGFP (CTRL), under the CMV promoter. Protein was then isolated using Thermo Scientific RIPA lysis buffer, quantified using BCA method, then subjected to western blotting as outlined above. ERβ and UCP1 protein expression were quantified relative to beta tubulin.

### Statistics

Within each sex, a 2 × 2 analysis of variance (ANOVA) was used to evaluate the effects of genotype (G = KO vs. WT) and treatment (T = CL vs. CTRL) and GxT interactions; 2 × 2 ANOVA was also used to determine effects of HFD (D = HFD vs. CHOW) and GxD interactions (prior to moving the HFD-fed animals through the intervention). In addition, using all groups, sex x treatment × genotype interactions were determined in order to determine whether genotype or treatment effects were sex-specific or sex-divergent. In other words, we used 2 × 2 × 2 ANOVA to determine whether there sex differences in how genotype affected responsiveness to CL. Outcome variables included physiological markers and protein expression. The results of the ANOVA are displayed below each figure, with the *p*-values for the main and interaction effects, as appropriate. All statistically significant interactions were followed by post-hoc Tukey’s tests and, if significant, are indicated by different letters on graphs as appropriate. When applicable, student t-tests were used to determine statistical significance between two individual groups. All data are presented as mean ± standard error of the mean (SEM). Significance was accepted at *p* < 0.05, except for when group numbers exceeded 15, in which cases *p* < 0.01 was considered statistically significant to reduce the likelihood of Type I error given the large sample size. All statistical analyses were performed with SPSS V25.0.

## Results

### Loss of ERβ deoxyribonucleic acid-binding domain does not influence response to high-fat diet or the ability of chemical ligand to improve body composition and energy balance

**FIGURE 2:**
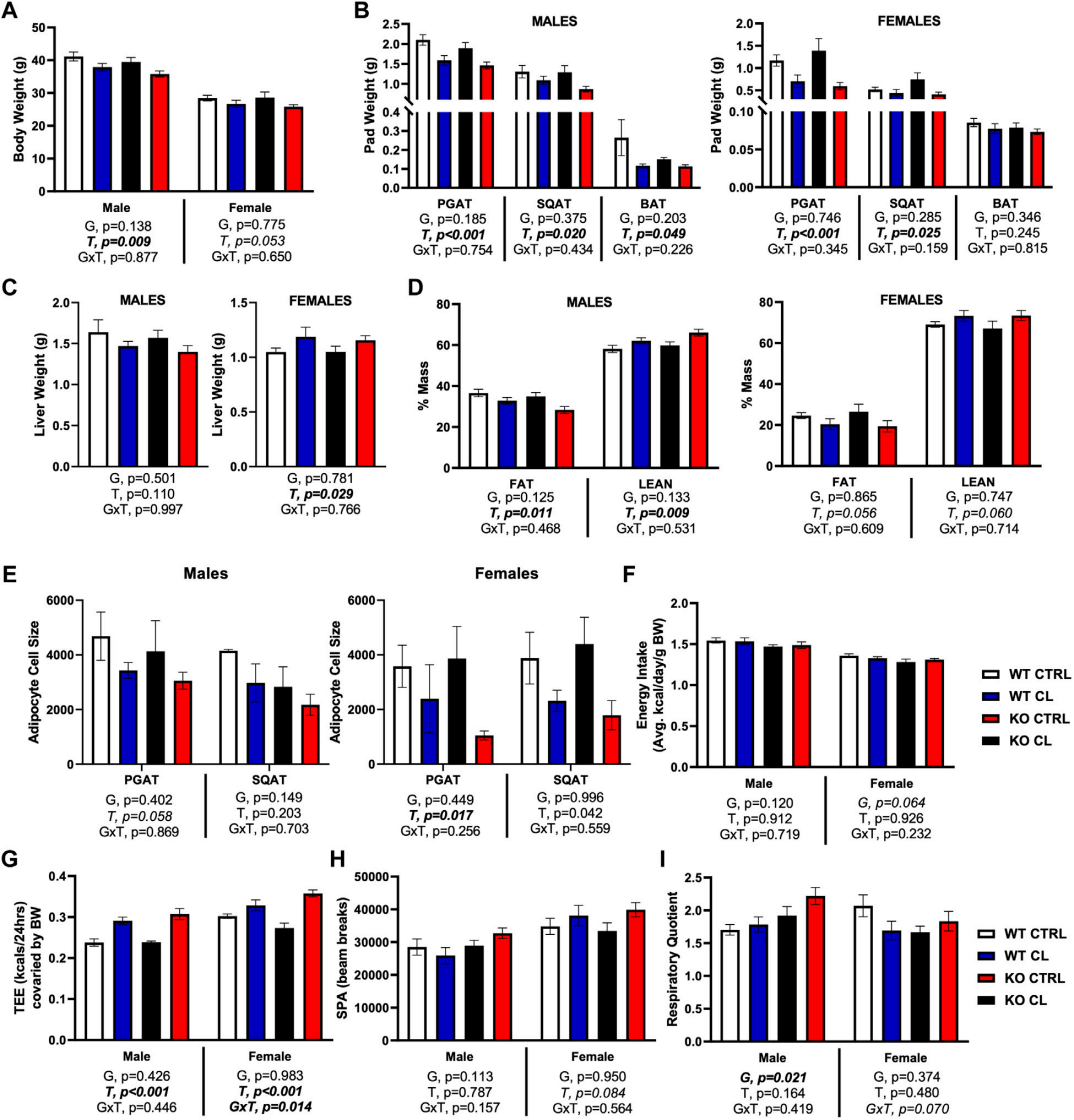
ERβ genomic activity is not required for CL-induced improvements in body composition and energy expenditure. Male and female C57BL/6J wild-type (WT) or Estrogen Receptor Beta (ERβ) DNA binding domain knockout (ERβ_DBD_KO) mice, all on HFD, were given 2-weeks CL injection (1ug/g body wight) or CTRL (saline) prior to body composition and energy expenditure assessment. **(A)** Final body weight. **(B)** Perigonadal (PGAT), subcutaneous (SQAT) and brown (BAT) adipose tissue pad weights. **(C)** Liver weight. **(D)** % Fat mass and % Lean mass. **(E)** PGAT and SQAT average adipocyte size. **(F)** Energy intake. **(G)** Total energy expenditure. **(H)** Spontaneous physical activity. **(I)** Respiratory quotient (RQ). All data are presented as mean ± SEM. *N* = 8–11 per group. Main effects of genotype (G) and CL treatment (T) and genotype by treatment interactions (GxT) for each sex were determined by 2 × 2 ANOVA and are presented below the respective figures. Post hoc Tukey’s tests were used to identify individual group differences in cases where significant interactions were found. *p*-value<0.05 accepted as significant.

We first show in Supplementary Figure S2 that there were no genotype differences in body weight, fat pad weights, or liver weight before or after HFD feeding. In both genotypes and in both sexes, HFD increased body weight (D, *p* < 0.001) (Supplementary Figure S2A), WAT pad weights (D, *p* < 0.001, *both depots*) (Supplementary Figure S2B), and liver weight (D, *p* = 0.043) (Supplementary Figure S2C), but had no effect on BAT pad weight. In our prior publication (Queathem Cells), we report on sex differences in how CL affects systemic and adipose tissue-specific metabolism using the WT mice used here as controls. Thus, in this study, we focus on the effects of genotype, as well as sex differences in how genotypes responded to CL. When the data were analyzed as a whole (males and females combined), CL’s ability to reduce HFD-induced body weight gain did not reach statistical significance (T, *p* = 0.096, *trend*). However, as shown in Figure 2, (Figure 2A), CL did have a significant body weight reducing effect in males, but was only trending in females. CL did reduce PGAT (T, *p* < 0.001) and SQAT (T, *p* < 0.03) pad weights in both sexes (Figure 2B). When sexes were combined, CL did not significantly reduce BAT pad weight (T, *p* = 0.072), yet when analyzed separately, did significantly reduce BAT in males. In terms of liver weight, there was no significant effect of CL overall, yet the sexes responded differently. CL tended to reduce liver weight in males and tended to increase liver weight in females, a modest but significant increase (Figure 2C). Overall, CL tended to improve body composition by decreasing % fat mass (T, *p* = 0.021) and increasing % lean mass (T, *p* = 0.018); but when sexes were analyzed separately, effects in males were significant whereas effects in females were only trending (Figure 2D). All of those effects were observed in both genotypes, with no GxT interactions, indicating that loss of ERβ DBD did not affect CL-mediated improvements in body composition in either sex. CL tended to reduce mean cell size in both PGAT and SQAT in both sexes and genotype did not affect cell size or CL’s effect on cell size (Figure 2E). When the entire cohort of animals were analyzed together, CL tended to increase relative energy intake (T, *p* = 0.027), but this effect did not reach statistical significance in either sex when analyzed separately (Figure 2F). Total energy expenditure (TEE) covaried for body weight increased with CL when sexes were combined (T, *p* < 0.001), an effect that remained significant when sexes were analyzed separately. Interestingly, this effect on TEE tended to be stronger in the ERβ_DBD_KO (GxT, *p* = 0.021 for sexes combined), an effect that was driven by the females (Figure 2G) (NEED TO SEE IF SEX INT EXISTS HERE) CL did not affect cage spontaneous physical activity (Figure 2H). When sexes were analyzed together, CL tended to decrease respiratory quotient (RQ) in the WT, whereas it tended to have the opposite effect in the ERβ_DBD_KO (GxT, *p* = 0.044) (Figure 2I). However, this trend was driven by the females, since when broken up by sex, a GxT interaction was not observed in the males, whereas there was a trend toward an interaction in females (*p* = 0.07). In summary, loss of ERβ_DBD_ (i.e., loss of classical genomic ERβ signaling) did not impact response to HFD, but sensitized female mice to the CL-induced increase in TEE.

### Chemical ligand improves glucose tolerance in both genotypes, but ERβ_DBD_KO females have a unique non-esterified fatty acids response

**FIGURE 3:**
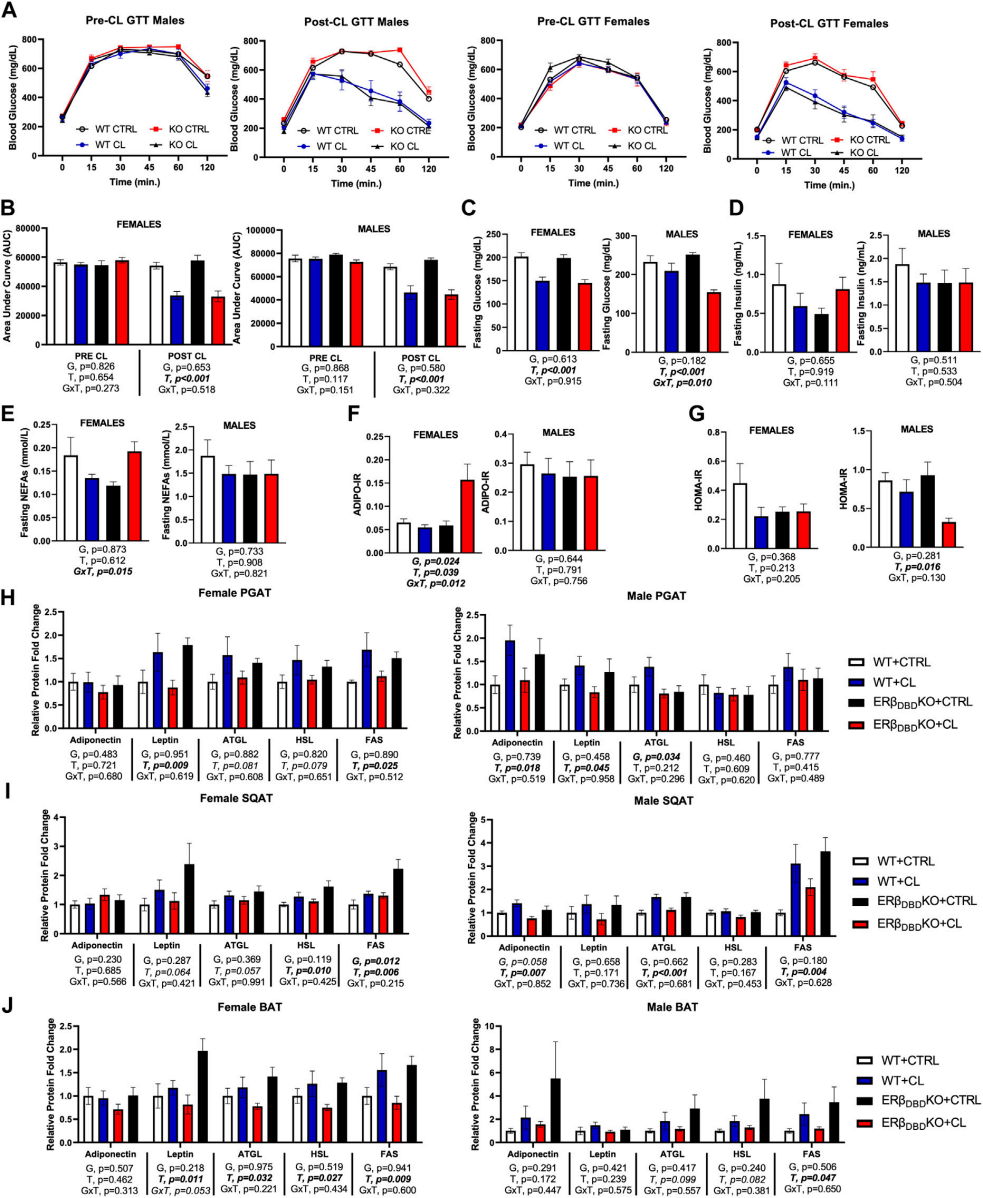
ERβ genomic activity is not required for CL-induced improvements in glucose homeostasis, or the augmentation of basal lipolytic response. Male and female C57BL/6J wild-type (WT) or Estrogen Receptor Beta (ERβ) DNA binding domain knockout (ERβ_DBD_KO) mice, all on HFD, were given 2-weeks CL injection (1ug/g body wight) or CTRL (saline). Glucose tolerance tests (GTT) were performed and blood biomarkers were measured to assess the effect of CL on glucose homeostasis. **(A)** GTT response graphs before and after CL treatment. **(B)** GTT area under curve (AUC) before and after CL treatment. **(C)** Fasting blood glucose. **(D)** Fasting blood insulin. **(E)** Fasting blood non-esterified fatty acids (NEFAs). **(F)** Adipose tissue insulin resistance (ADIPO-IR) = fasting NEFA concentration (mmol/L) x fasting insulin concentration (pmol/L). **(G)** Homeostatic Model Assessment for Insulin Resistance (HOMA-IR) = fasting insulin (microU/L) x fasting glucose (nmol/L)/22.5. At sacrifice, perigonadal (PGAT), subcutaneous (SQAT) and brown (BAT) adipose tissue depots were collected and the expression of adipokines (e.g., adiponectin and leptin) and lipid metabolic markers (e.g., ATGL, HSL, FAS) were measured via western blot and expressed relative to beta actin. ATGL (Adipose triglyceride lipase); HSL (Hormone sensitive lipase); FAS (Fatty acid synthase). **(G)** PGAT adipokine and lipid protein expression. **(H)** PGAT adipokine and lipid protein expression. **(I)** SQAT adipokine and lipid protein expression. **(J)** BAT adipokine and lipid protein expression. All data are presented as mean ± SEM relative to the WT CTRL. *N* = 7–14 per group. Main effects of genotype (G) and CL treatment (T) and genotype by treatment interactions (GxT) were determined by 2 × 2 ANOVA and are presented below the respective figures. Post hoc Tukey’s tests were used to identify individual group differences in cases where significant interactions were found. *p*-value<0.05 accepted as significant.

Loss of ERβ DBD did not affect glucose response or insulin sensitivity, nor did it have any marked effect on CL’s insulin-sensitizing effects in either males or females. Area under the curve (AUC) during glucose tolerance test (GTT) (Figures 3A,B) and fasting glucose (Figure 3C) were both significantly reduced by CL in both genotypes and sexes, as expected. Among males only, ERβ_DBD_KO were more sensitive to CL’s ability to reduce fasting glucose (GxT, *p* = 0.01). Fasting insulin was not affected by genotype or treatment in either sex (Figure 3D). In contrast, the two genotypes tended to respond differently to CL in terms of fasting NEFAs when sexes were analyzed together (GxT, *p* = 0.036); however, when investigated separately by sex, this was driven by the females, who experienced a significant GxT interaction (*p* = 0.015) such that CL decreased NEFAs in the WT and increased NEFAs in theERβ_DBD_KO (Figure 3E). However, it is important to point out that the females also had ∼10-fold lower NEFAs to begin with, so even with the CL-induced increase, levels were still much lower than the males. Similarly, genotypes responded differently to CL’s effect on ADIPO-IR (GxT, *p* = 0.020) when sexes were analyzed together, an effect driven by the females (Figure 3F). The effect of CL to improve HOMA-IR was significant for the entire cohort, and effect largely driven by the males, likely because the males were more insulin resistant to begin with and had greater room for improvement (Figure 3G). Collectively, these data show that loss of ERβ DBD (i.e., classical genomic ERβ signaling) does not robustly influence systemic glucose tolerance, and is not required for the beneficial effects of CL on glucose homeostasis. However, it does play a modulatory role in CL responsiveness. As shown previously (Queathem), males were more metabolically dysfunctional. Here, we show that KO males were more sensitive to CL’s protective effects, likely due to their greater room for improvement. On the other hand, KO females had a divergent response to CL on fasting NEFAs.

To better understand the impact of genotype on CL-mediated effects on adipose tissue metabolism, we looked at several metabolic protein markers in WAT and BAT depots (Figures 3H–J). When sexes were combined, in both genotypes, CL increased fatty acid synthase (FAS), adipose triglyceride lipase (ATGL) and leptin (T, *p* < 0.05, *all*), in PGAT, SQAT, and BAT, similar to previous reports (Queathem)**.** Hormone sensitive lipase (HSL) was also increased in SQAT (T, *p* = 0.005). Collectively, these data demonstrate that there were no genotype differences in how CL affected those adipose tissue markers when males and females were combined. When broken up by sex, very subtle genotype differences were observed. In males, there was a main effect of genotype on PGAT ATGL such that KOs had lower levels; and in females, there was a main effect of genotype on SQAT FAS (higher levels in KOs).) The effects of CL on AMP Kinase (AMPK) were similar to previous findings and not affected by genotype (Supplementary Figure S3).

### Loss of the ERβ deoxyribonucleic acid-binding domain activity may reduce adipocyte mitochondrial O_2_ consumption in the basal state, an effect rescued by chemical ligand

**FIGURE 4:**
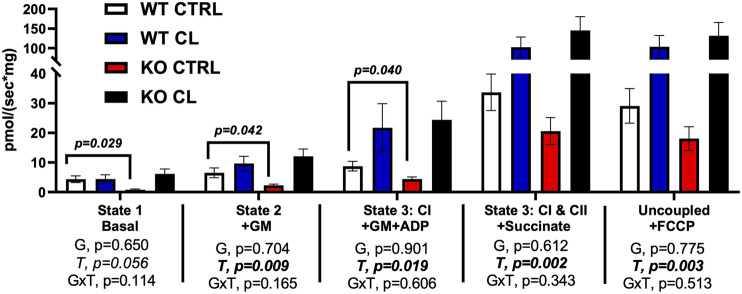
Adipocytes from mice lacking ERβ genomic activity have lower basal mitochondrial respiration, an impairment completely rescued by CL treatment. Male and female C57BL/6J wild-type (WT) or Estrogen Receptor Beta (ERβ) DNA binding domain knockout (ERβ_DBD_KO) mice, all on HFD, were given 2-weeks CL injection (1ug/g body wight) or CTRL (saline), then perigonadal mitochondrial respiration (i.e., oxygen consumption) was measured via Ouroboros. All data are presented as mean ± SEM. *N* = 8–13 per group. Main effects of genotype (G) and CL treatment (T) and genotype by treatment interactions (GxT) were determined by 2 × 2 ANOVA and are presented below the respective figures. T-tests comparing WT and ERβ_DBD_KO controls was also assessed and displayed on graph were significant. *p*-value<0.05 accepted as significant for all tests.

Adipocytes were harvested from a subset of male and female mice (PGAT depot) and assessed *ex vivo* for substrate-driven oxygen consumption. Insufficient samples from each sex were acquired in order for sex differences to be determined, so the sexes were combined for this experiment. As shown in Figure 4, there were no main genotype effects on mitochondrial respiration (G, *p* > 0.05, *all*). However, when compared to WT CTRL mice, ERβ_DBD_KO CTRL mice tended to have lower basal, GM, and GM + ADP O_2_ consumption. CL completely rescued these deficits. CL significantly increased O2 consumption in all stimulated states (T, *p* < 0.020, *all*), and there was a strong trend detected in the basal state (T, *p* = 0.056, *trend*). In summary, adipocytes harvested from mice lacking classical genomic ERβ signaling tend to show lower mitochondrial O_2_ consumption at rest, which is completely rescued with CL. These results may suggest enhanced metabolic flexibility in the adipocyte at the level of the mitochondria, but additional studies are required before definitive conclusions can be made.

### Loss of ERβ deoxyribonucleic acid-binding domain does not affect chemical ligand-mediated increase in mitochondrial proteins, but increases white adipose tissue sensitivity to UCP1 induction

**FIGURE 5:**
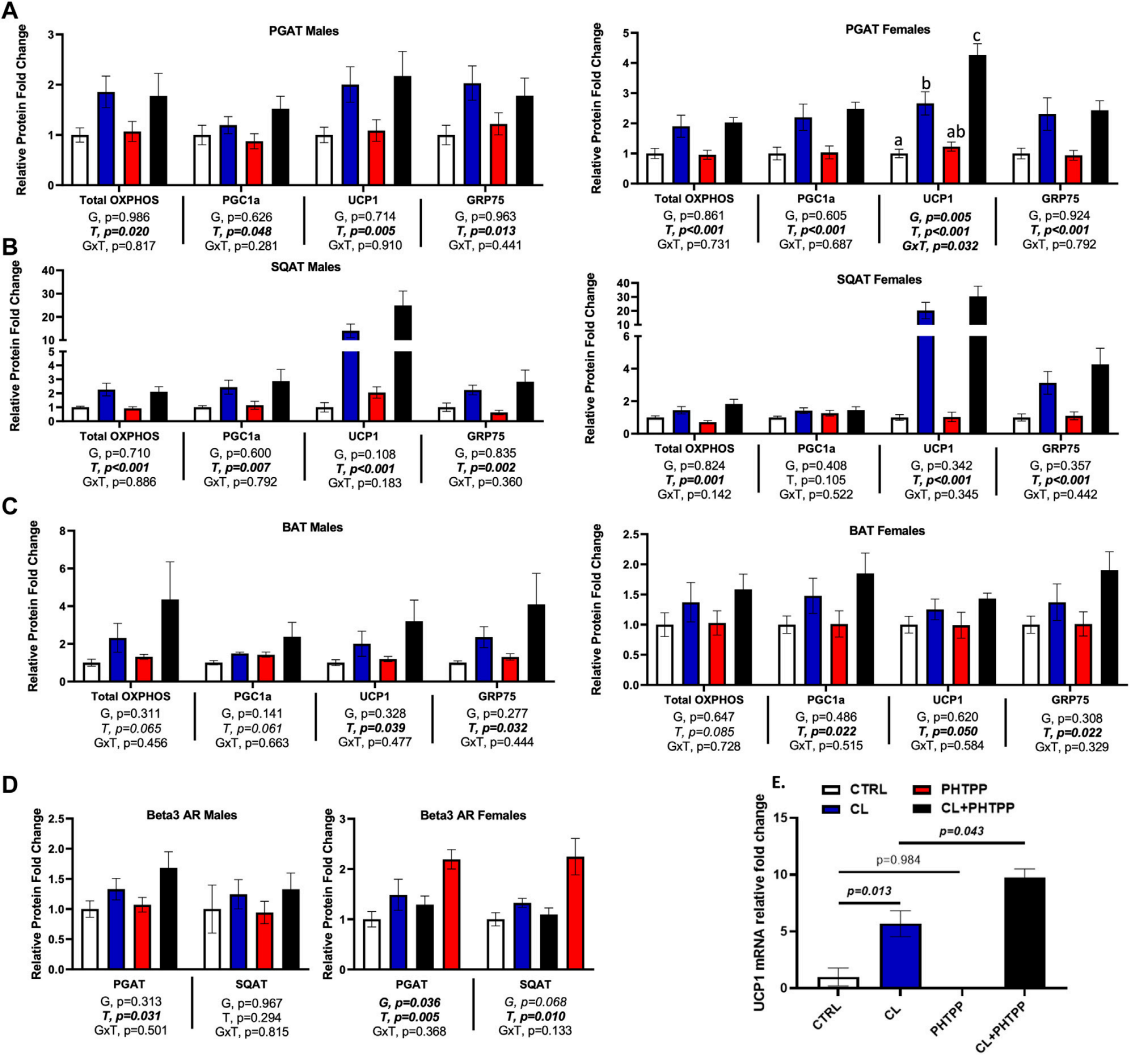
*In vivo* and *in vitro*, suppression of ERβ genomic activity increases WAT sensitivity to CL-induced UCP1. Male (*left panels*) and female (*right panels*) C57BL/6J wild-type (WT) or Estrogen Receptor Beta (ERβ) DNA binding domain knockout (ERβ_DBD_KO) mice, all on HFD, were given 2-weeks CL injection (1ug/g body wight) or CTRL (saline). Perigonadal (PGAT), subcutaneous (SQAT) and brown (BAT) adipose tissue depots were collected and the expression of mitochondrial proteins were measured via western blot. Total OXPHOS (Combined markers for complexes 1-5 of the oxidative phosphorylation proteins); PGC1a (Peroxisome Proliferating Receptor Gamma Coactivator 1 alpha protein); UCP1 (mitochondrial uncoupling protein 1); GRP75 (Glucose-response protein 75); beta actin uses as normalizer protein. **(A)** PGAT protein expression **(B)** SQAT protein expression **(C)** BAT protein expression **(D)** β3-adrenergic receptor (β3AR) protein expression; relative to beta actin **(E)** Primary adipocytes harvested from PGAT and SQAT samples extracted from non-treated male C57BL6J wild-type mice were treated *in vitro* with either 1 uM CL, 1 uM of the ERβ selective antagonist, PHTPP (*i.e., chemical inhibitor of ERβ genomic activity*) or 1 uM of the combination of CL and PHTPP. After 24 h treatment, Ucp1 mRNA induction was measured using qRT-PCR relative to beta-actin; forward primer (5′-3′): CAC​GGG​GAC​CTA​CAA​TGC​TT; reverse primer (5′-3′): ACA​GTA​AAT​GGC​AGG​GGA​CG; ncbi ref seq: NM_009463.3. All data are presented as mean ± SEM, relative to the WT CTRL. *N* = 7-9 per group (*in vivo*) and 8 per group (*in vitro*). Main effects of genotype (G) and CL treatment (T) and genotype by treatment interactions (GxT) were determined by 2 × 2 ANOVA and are presented below the respective figures. *p*-value<0.05 accepted as significant for all tests. Lower case letters are used to distinguish statistically different groups as determined by Tukey’s post-hoc tests, which were conducted only in cases were statistically significant interactions were observed.

Figure 5 summarizes how genotype affected CL-mediated adipose tissue browning responses and the figures are organized by sex. When sexes were analyzed together, there were no genotype differences in the response to CL on individual mitochondrial OXPHOS complex protein expression in PGAT, SQAT and BAT, or in total OXPHOS expression; similar to previous findings, CL increased OXPHOS protein, as well as PGC1a, UCP1 and GRP75 in all three depots (T, *p* < 0.015, *all*). Data for individual OXPHOS complexes (C1-C5) can be found in Supplementary Figure S3. There were genotype differences in both PGAT and SQAT, such that ERβ_DBD_KO mice had elevated levels of UCP1; however there were no such genotype differences in the mitochondrial biogenesis marker, PGC1a, or the newly identified browning marker, GRP75. There were also no genotype differences found in BAT. Interestingly, ERβ_DBD_KO mice tended to be more responsive to CL-induced UCP1 in SQAT compared to WT (GxT, *p* = 0.017). However, when separated by sex, this effect was no longer true in either sex. Although the effect appeared to be more driven by males. In PGAT, female KOs were more responsive to CL-induced UCP1. In our previously published work, we demonstrated that females were more sensitive to CL-induced UCP1 compared to males; here, we show that suppression of ERβ DNA binding activity further heightens this effect in females. Given the increased sensitivity to CL in the female ERβ_DBD_KO mice, the expression of the receptor for CL, the beta 3 adrenergic receptor (β3AR) was assessed. Similarly, and only among females, KOs had greater PGAT β3AR expression. When sexes were analyzed together, the level of β3AR still tended to be higher in the ERβ_DBD_KO mice compared to WT (G, *p* = 0.022) in PGAT (driven by the effect in females noted above), but did not reach statistical significance in SQAT. These effects mirrored the UCP1 data, where the increase was driven by the females.

Next, we tested *in vitro* the mechanism by which non-classical genomic functions of ERβ may be involved in the CL-mediated increase in UCP1. Primary adipocytes from WT mice were treated with an ERβ selective inhibitor (PHTPP), known to inhibit ERE-dependent (i.e., DBD-dependent) functions of ERβ (Compton et al., 2004; Hsu et al., 2014), alone or in combination with CL. In parallel to the *in vivo* observations in females, PHTPP synergized with CL to increase UCP1 mRNA in primary adipocytes (*p* = 0.043) (Figure 5E). These data demonstrate for the first time *in vitro*, that ERβ specific ligands influence the response to CL, and further support the *in vivo* evidence demonstrating that inhibition of classical genomic ERβ signaling (e.g., modeled by the ERβ_DBD_KO) enhances the CL-mediated induction of UCP1, at least in females, although it should be noted that the primary adipocytes used in the *in vitro* studies were from male mice. Overall, these findings suggest that promoting novel non-classical ERβ activities in adipocytes may enhance cellular and mitochondrial activity through modulation of UCP1.

### Depot-specific adipose tissue estrogen receptor expression differences

**FIGURE 6:**
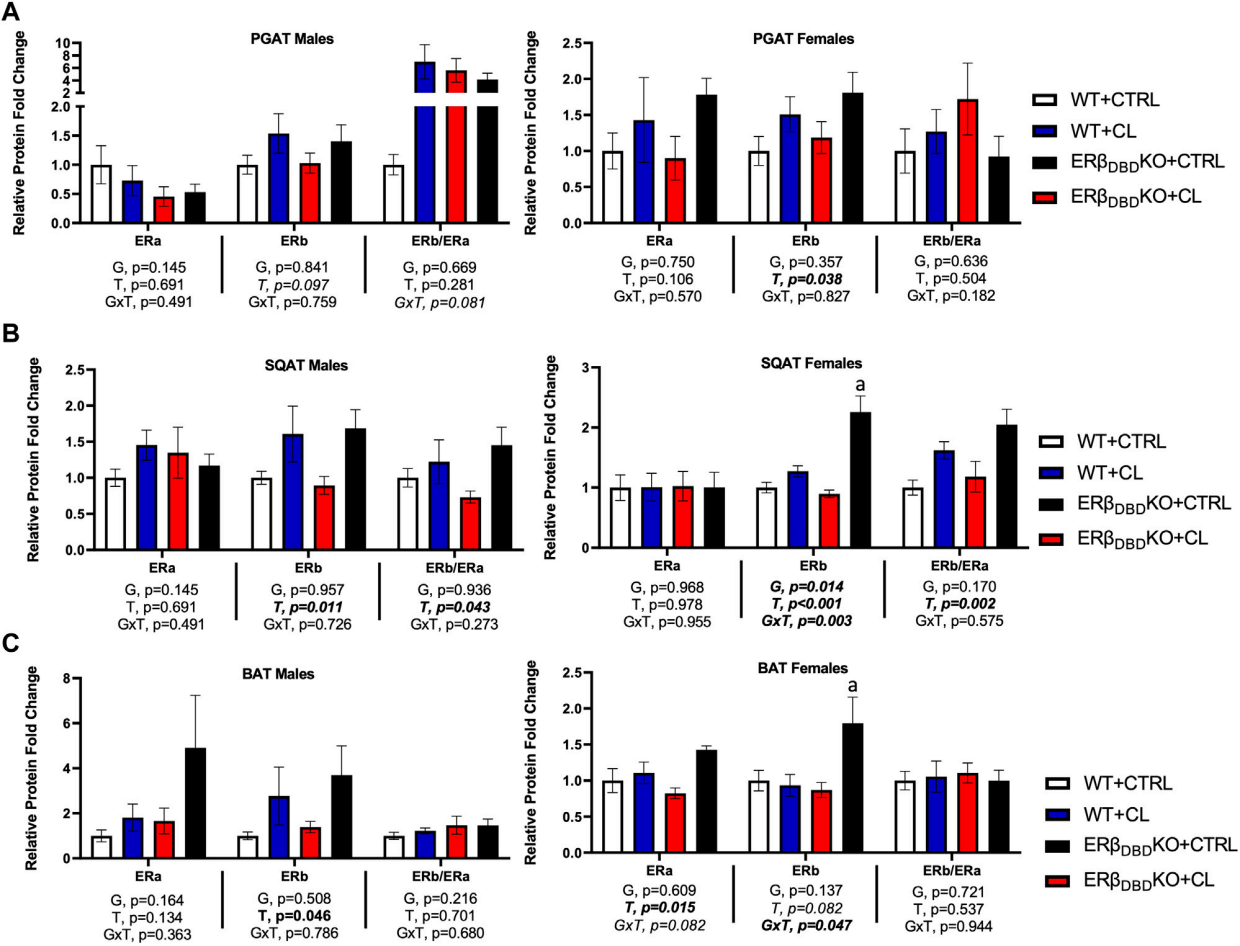
Independent of ERβ DBD, CL increases total ERβ protein expression in WAT. Male and female C57BL/6J wild-type (WT) or Estrogen Receptor Beta (ERβ) DNA binding domain knockout (ERβ_DBD_KO) mice, all on HFD, were given 2-weeks CL injection (1ug/g body wight) or CTRL (saline). Perigonadal (PGAT), subcutaneous (SQAT) and brown (BAT) adipose tissue depots were collected and the expression of estrogen receptor alpha (ERα), estrogen receptor beta (ERβ) and the ERβ to ERα ratio (ERβ/ERα) was assessed via western blot relative to beta tubulin. **(A)** PGAT total and relative ER expression. **(B)** SQAT total and relative ER expression. **(C)** BAT total and relative ER expression. All data are presented as mean ± SEM, relative to the WT CTRL; *N* = 7–9/group. Main effects of genotype (G) and CL treatment (T) and genotype by treatment interactions (GxT) were determined by 2 × 2 ANOVA and are presented below the respective figures. *p*-value<0.05 accepted as significant for all tests. Lower case letters are used to distinguish statistically different groups as determined by Tukey’s post-hoc tests, which were performed in instances where GxT interactions occurred.

In Figure 6, ER protein expression was assessed across adipose tissue depots. With sexes combined, there were no genotype differences in ERα or ERβ protein expression in PGAT, SQAT, or BAT. Furthermore, CL had no main effect on ERα expression in any fat depot. However, confirming our previous findings (Clookey et al., 2019; Queathem et al., 2021), CL increased ERβ protein expression across depots (T, *p* < 0.05, *all*), effects true in both sexes. Interestingly, when sexes were combined, CL was more effective at increasing ERβ protein in SQAT in the ERβ_DBD_KO compared to WT (GxT, *p* < 0.001), which paralleled the heightened UCP1 response to CL in the ERβ_DBD_KO. However, when separated by sex, this effect was only significant among females, where KOs were more sensitive to CL-induced ERβ induction in both SQAT and BAT. However, independent of genotype, CL significantly increased the relative abundance of ERβ (i.e., ERβ to ERα ratio) in SQAT (T, *p* = 0.002)**,** which remained significant when analyzed in both males and females.

### Full ERβ function is required for UCP1 expression *in vitro*

**FIGURE 7:**
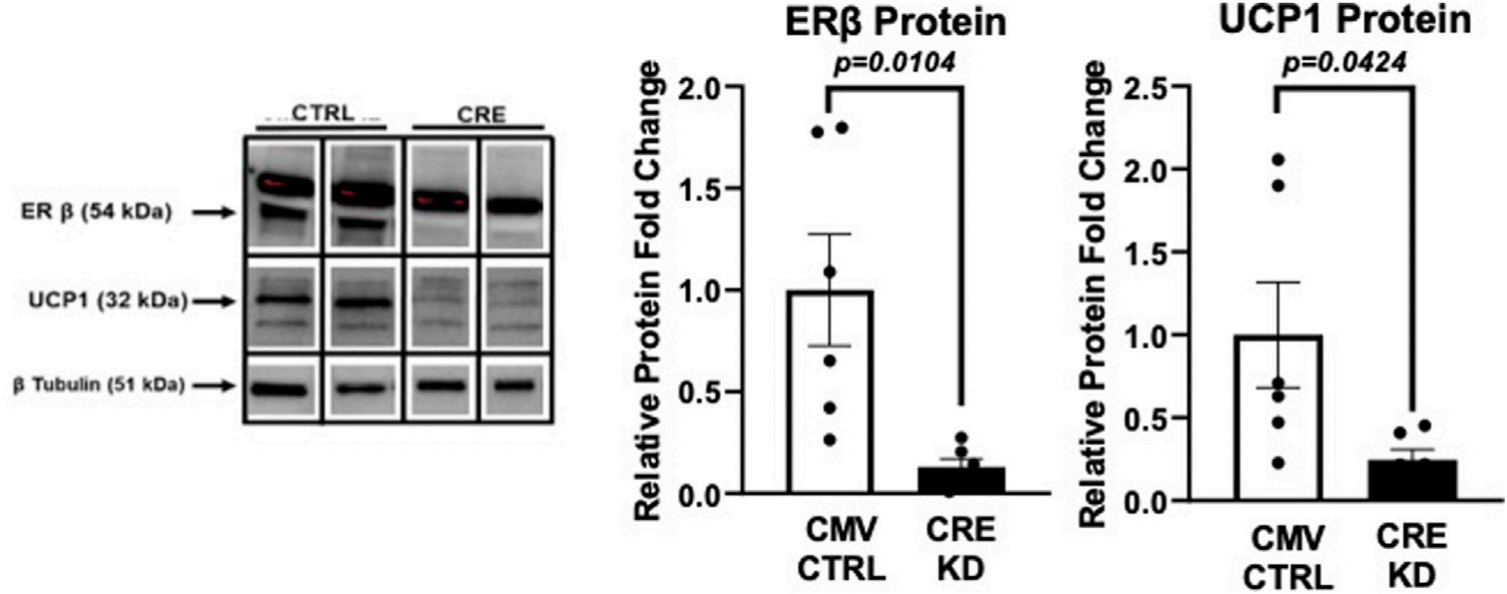
*Ex vivo* knockdown of ERβ suppresses UCP1. Primary adipocytes isolated from PGAT and SQAT harvested from one male Esr2-loxP floxed mouse^cre+/-^ were transfected 14 days after induction with 1.0 × 10^8^ plaque-forming units/ml media recombinant adenovirus (Vector Biolabs, Malvern, PA) containing Cre-recombinase (CRE), or eGFP (CTRL), under the CMV promoter, for 24 h to decrease expression of ERβ, *encoded by esr2*. Western blotting was performed to assess protein expression levels of ERβ and the mitochondrial uncoupling protein 1 (UCP1) relative to beta tubulin. All data are presented as mean ± SEM *N* = 6/group; *p*-value<0.05 accepted as significant.

Given the emergent relationship between WAT ERβ and UCP1, we next tested whether full ERβ is required for UCP1 expression *in vitro.* Using primary adipocytes harvested from a male Esr2-loxP floxed mouse^cre +/-^ administration of CRE-adenovirus was used to knock down the expression of ERβ protein *in vitro*, which proved successful (*p* = 0.0104) (Figure 7). Remarkably, knock down of ERβ led to a comparable knock down in UCP1 protein expression (*p* = 0.04), demonstrating for the first time *in vitro*, the necessity of full ERβ protein in regulating adipocyte UCP1.

## Discussion

Estrogen (E2)-sufficient females demonstrate protection against insulin resistance and white adipose tissue (WAT) metabolic dysfunction (Goossens et al., 2021). This protection likely involves WAT-specific E2 signaling. Indeed, E2 (or E2 analogs) affect adiposity, WAT distribution and the metabolic profile of adipocytes (Naaz et al., 2003; D’Eon et al., 2005). Mechanistically, the vast majority of work in this area has focused on E2 receptor subtype alpha (ERα), which is the feminizing subtype (Uenoyama et al., 2021) more densely expressed in female WAT. However, E2 influences body composition, fat distribution and metabolism in both sexes (Zidon et al., 2020; D’Eon et al., 2005; Kalyani et al., 2009) and the other main ER subtype, ERβ, is also expressed in adipose tissue, and there are not appreciable sex differences in its adipose tissue-specific expression levels (Queathem et al., 2021). Importantly, the adipose tissue-specific functions of ERβ are largely unknown and require further study as a potential target to improve adipocyte metabolism in both sexes, and in instances where ERα activation is not recommended.

The compound, CL 316,243 (CL) is a highly selective β3AR ligand known to enhance adipocyte mitochondrial metabolism, leading to weight loss and improved overall metabolism (Himms-Hagen et al., 1994; Yoshida et al., 1994; Ghorbani and Himms-Hagen, 1997; Kaisanlahti and Glumoff, 2019). CL has been shown to improve obesity-associated insulin resistance in other models, and we have shown this compound to be sufficient to rescue metabolic dysfunction in ERα null mice, a model of metabolic dysfunction due to loss of E2 signaling (Clookey et al., 2019). We recently investigated sex differences in the metabolic effects of CL in the setting of diet-induced obesity in mice (Queathem et al., 2021) and demonstrated that both sexes are sensitive to the metabolic improvements induced by CL treatment. We went on to show sex and adipose tissue-depot specific differences in CL’s effects. An important finding of that paper was that CL significantly increased adipose tissue expression of ERβ, confirming our previous work (Queathem et al., 2021), and demonstrating that CL increases ERβ in both sexes. The purpose of this current study was to answer the following questions: 1) What role do the classical genomic functions of ERβ (*i.e., those directly requiring nuclear DNA binding domain (DBD* *) binding*) play in the systemic and adipose tissue responses to CL, including its ability to induce UCP1? 2) Are there sex differences in how these aspects of ERβ function affect systemic or adipose tissue-specific responses to CL? 3) Mechanistically, are these non-classical/genomic aspects of ERβ function required for CL-induced ERβ upregulation? We hypothesized that: 1) ERβ plays a necessary role in CL-mediated UCP1 responses, but its classical DBD-dependent genomic actions are not required for the effectiveness of CL. 2) Sex differences do not exist in how ERβ influences systemic or adipose tissue responses to CL. To this end, we used a genetic rodent model that lacks ERβ DBD, but retains functions of ERβ that do not require its DNA binding (ERβ_DBD_KO) (Clart et al., 2021), to investigate the role that non-classical signaling of ERβ may play in systemic physiology and adipocyte metabolism in the setting of diet-induced obesity, with an emphasis on its role in mediating WAT browning in response to CL. In addition, we used primary adipocytes to test *in vitro* whether non-classical functions of ERβ would enhance the effectiveness of CL, and finally, used Cre recombinase-mediated knock-down of ERβ *in vitro* to test the necessity of ERβ for adipocyte UCP1 expression.

While the classical genomic actions of ERα and ERβ have been the primary focus of metabolism research, the non-classical functions (i.e., functions that do not directly require nuclear binding to the DBD, the major pathway for classical gene expression regulation by ERs) have been largely ignored. Here, we demonstrate for the first time that DBD-dependent ERβ functions are not required for the well-established (Yoshida et al., 1994; Ghorbani and Himms-Hagen, 1997; MacPherson et al., 2014) beneficial effects of CL on body composition, glucose tolerance, and WAT browning in either sex. On the contrary, abolishing DBD-dependent ERβ actions either *in vivo* (i.e., ERβ_DBD_KO model) or *in vitro* (i.e., PHTPP treatment) led to *increased* sensitivity in CL-induced WAT browning (i.e., UCP1 expression), at least in female mice. This coincided with greater CL-induced increase in total energy expenditure and an altered RQ response in female ERβ_DBD_KO in particular. This heightened response to CL-mediated UCP1 induction was paralleled by an increased sensitivity to CL-induced ERβ protein expression, further supporting the strong relationship between ERβ and UCP1 in WAT that our lab has previously demonstrated in mice and humans (Clookey et al., 2019; Clart et al., 2021; Queathem et al., 2021). We now extend these findings to show this relationship exists even in a model that lacks classical genomic functions of ERβ, indicating that this relationship does not require classical genomic activity of ERβ. Collectively, these findings lead us to posit that promoting the non-classical functions of ERβ may be a therapeutic strategy to increase mitochondrial metabolism in adipocytes and, in so doing, improve systemic metabolic health in the setting of obesity.

We first demonstrate that deleting the DBD of ERβ did not influence body composition or alter the response to HFD-feeding in either male or female mice. This was not surprising since other animal models totally lacking ERβ have demonstrated this lack of phenotype as well (Krege et al., 1998; Antal et al., 2008). Consistently, the ERβ_DBD_KO mice consumed similar calories and expended similar energy per Gram body weight compared to WT littermate controls. There were also no genotype differences in glucose tolerance. In both genotypes, CL did not affect energy intake or physical activity, yet increased total energy expenditure, demonstrating that the improvements in energy balance were due to increases in basal metabolic rate, consistent with the known effects of CL to promote fat oxidation (Himms-Hagen et al., 1994; Yoshida et al., 1994; Ghorbani and Himms-Hagen, 1997). Despite not affecting CL’s ability to improve body composition in either sex, genetic ablation of ERβ DBD did modestly affect the metabolic response to CL, an effect apparently driven by female mice. That is, KO females were slightly more responsive to CL-induced increase in total energy expenditure, which coincided with divergent NEFA responses between WT and KO females. Whereas WT females experienced a decrease in circulating fasting NEFAs, the ERβ_DBD_KO mice experienced an increase. This may be explained by an elevation in CL-mediated lipolysis in female KOs, but since adipocyte lipolysis was not directly measured, this cannot be definitively concluded. Nonetheless, this divergent metabolic response to CL associated with both heightened sensitivity to adipose tissue UCP1 induction and heightened sensitivity to CL-induced ERβ. At least in the KO mice, it can be assumed that this induced protein was the mutated version lacking DBD-specific actions.

In terms of CL’s effects on glucose metabolism, ERβ DBD was not required for those benefits to occur. However, modest sex-specific genotype effects were observed, suggesting that lack of ERβ DBD did affect CL’s glucose regulatory role, at least in a minor way. It is well established (Kuk and Ardern, 2010; Tournissac et al., 2020), and we recently confirmed, that males are more susceptible to obesity-induced metabolic dysfunction. Recently, we demonstrated that CL effectively improves insulin resistance and adipose tissue metabolism in males (Queathem et al., 2021). Here, we found that KO males, who are more insulin resistant to begin with, were more sensitive to CL-induced improvements, demonstrated by the HOMA-IR index. Genotype did not affect CL’s ability to restore insulin sensitivity in females, but this may have been attributed to the fact that females did not experience as significant an impairment in glucose metabolism. That is, there was more ‘room for improvement’ in males. In females, the divergent NEFA response between genotypes coincided with a divergent effect of CL on ADIPO-IR, an index of adipocyte-specific insulin resistance. That is, whereas WT tended to see a reduction with CL, KO responded in the opposite direction. This may seem counterintuitive, but it should be noted that female adipose tissue is significantly more insulin sensitive than male adipose tissue by a magnitude of ∼10. That is, while absence of ERβ DBD appeared to increase Adipo-IR among females, driven by the higher NEFA concentrations, even the highest Adipo-IR level was about 10-fold lower than that of the most insulin sensitive males. Thus, the physiological relevance of these genotype differences among females is uncertain. Given that levels remained in the healthy range in all groups of females, we do not consider this an adverse physiological response.

To further interrogate the mechanism for this augmented metabolic response in female KOs, and heightened sensitivity to insulin-sensitizing effects of CL among males, adipose tissue mitochondrial-related proteins were measured and compared, along with adipokines and lipolysis-related proteins. Among females, ERβ_DBD_KO mice had increased β3AR protein expression in PGAT, suggesting that this WAT depot may be more sensitive to CL-mediated lipolysis, perhaps explaining their increased NEFA response. This may suggest that elimination of classical genomic ERβ signaling may facilitate WAT lipolysis, leading to increased fat oxidation upon lipolytic stimulation. This is supported by the enhanced CL-mediated increase in energy expenditure observed in the female KO mice. Also in the ERβ_DBD_KO females, SQAT FAS protein was increased. In our prior publication, we showed across depots and in both sexes that CL increased FAS expression, suggesting an increase in lipolytic turnover induced by CL. That is, CL not only increases oxidation of lipids, but increases the metabolic processing of lipids. Here, we show that this response may be increased in the KO females. Key evidence supporting this is that female KOs were significantly more sensitive to CL-induced WAT UCP1 induction (i.e., browning), especially in PGAT (Figure 5), the depot we previously showed to be more responsive to browning in females.

Mechanistically, we posit that this increased sensitivity to browning is due to heightened non-classical signaling activities of ERβ involving its ability to localize to adipocyte mitochondria and directly affect mitochondrial activity. In order to more closely assess how adipocyte mitochondrial energy expenditure was affected by CL, we measured adipocyte O_2_ consumption in freshly isolated adipocytes harvested from the PGAT depot of male and female WT and KO mice treated with CL versus vehicle control. Due to the amount of adipose tissue required in order to isolate sufficient adipocytes for those *ex vivo* experiments, we had to pool our male and female adipocytes and thus were not able to examine sex differences in adipocyte mitochondrial metabolism. Future studies need to determine sex differences. In examining adipocyte metabolism among the WT and KO non-CL treated mice (via independent samples t-tests), we found that the KOs had suppressed basal adipocyte mitochondrial O_2_ consumption. However, CL completely rescued this deficit in O_2_ consumption, perhaps indicating that the KO adipocytes had increased metabolic flexibility. This altered fuel partitioning in adipocytes may have contributed to the increase in energy expenditure observed in the female KOs following CL treatment. Thus, classical genomic signaling of ERβ may influence adipocyte mitochondrial function, yet is not required for the mitochondria-specific effects of CL.

*Relationship between UCP1 and ERβ?* Given that UCP1 is known to buffer mitochondrial ROS production (Oelkrug et al., 2014; Chouchani et al., 2016), and associates with enhanced metabolic health and lower inflammation (Winn et al., 2017; Mills et al., 2021), enhanced CL-induced UCP1 is likely a metabolically advantageous adaptation in the ERβ_DBD_KO. These data support an important relationship between ERβ and UCP1 in WAT, implicating ERβ as playing a role in WAT mitochondrial activity in part via modulation of UCP1. Our previous work in rodents (Queathem et al., 2021) and humans (Porter et al., 2020) has already established a highly significant, consistent, and strong correlation between these proteins in WAT. This current study demonstrates that this relationship between UCP1 and ERβ exists even when ERβ is mutated to be incapable of binding EREs. To further probe that relationship between UCP1 and ERβ, *in vitro* studies were performed using full ERβ knock-down, which was accomplished by treating adipocytes harvested from Esr2-flox mice with Cre-recombinase. In that experiment (Figure 7), we demonstrate for the first time that full ERβ protein is indeed required for UCP1 expression in adipocytes*.* Combined with the *in vivo* evidence from the ERβ_DBD_KO model, these data collectively demonstrate that: 1) there is an important link between ERβ and UCP1 in adipose tissue, and 2) this link is mediated through an ERβ-DBD-independent mechanism. Evidence is that, in investigating whether ERβ-DBD was requisite for the relationship between ERβ and UCP1 by determining whether the ERβ_DBD_KO mice would also experience an increase in ERβ upon CL stimulation, we observed a *heightened* sensitivity to CL-induced ERβ increase in the mice lacking classical genomic functions of ERβ, coinciding with increased responsiveness to CL-induced UCP1. Future studies are necessary to definitively determine the physiological relevance of CL-induced ERβ, but we take this as evidence to support that CL facilitates non-classical functions of ERβ, possibly specific to adipocyte mitochondria. In support, ERβ has been shown to localize to mitochondria and directly affect mitochondrial oxidative phosphorylation in other types of cells (Chen et al., 2004; Chen et al., 2009; Liao et al., 2019; Song et al., 2019).

## Perspective and limitations

E2 is known to regulate mitochondrial biogenesis (Klinge, 2008; Chen et al., 2009; Klinge, 2017) and ROS production (Burris and Krishnan, 2005; Razmara et al., 2007; Razmara et al., 2008; Liao et al., 2019). Further, the role and regulation of UCP1 in buffering mitochondrial ROS is well-documented (Shabalina et al., 2006; Chouchani et al., 2016; Jia et al., 2019) and conserved across species (Oelkrug et al., 2014). Our group and others have demonstrated that sex plays a major role in WAT browning (Kim et al., 2016; Queathem et al., 2021) and there is a growing body of evidence supporting a functional link between E2 and uncoupling proteins (Pedersen et al., 2001). Over the past decade, ERβ selective ligands have been shown to alleviate HFD-induced metabolic dysfunction (Yepuru et al., 2010; Ponnusamy et al., 2017; González-Granillo et al., 2019a; González-Granillo et al., 2019b) and induce WAT browning (Miao et al., 2016), strikingly similar to the effects of CL. Mechanistically, our present work supports a necessary relationship between ERβ and UCP1, and indicates that the classical genomic actions of ERβ are not required for this relationship, raising the possibility that ERβ ligands may be activating adipocytes through non-classical (e.g., rapid signaling) mechanisms. This is not an unreasonable assumption since ERβ has been shown to mediate important non-genomic actions in other cell types (Chambliss et al., 2002; Zhao and Brinton, 2007; Gingerich and Krukoff, 2008)^,^ (Maneix et al., 2015). Our study adds adipose tissue metabolism to that list of processes affected by such “non-classical” ERβ pathways. That being said, and in keeping with the essentiality of ERβ in regulating UCP1, the role that ERβ plays in regulating UCP1 is apparently complex and likely dependent upon a variety of physiological factors. In a previous study, we showed that female ERβ_DBD_KO mice in the ovariectomized state are completely *resistant* to exercise-induced adipose tissue UCP1 induction, unlike WT control mice (Clart et al., 2021). While this appears contradictory to the present results, which indicate that the ERβ DBD is *not required* for drug-induced UCP1 increase and its suppression may actually *increase* drug-induced UCP1responses, two important differences in these studies may have resulted in those divergent findings: 1) the current study used mice in the ovary-intact state and 2) the CL stimulus is very different from the exercise stimulus; thus, the mechanisms by which exercise and CL induce adipose tissue UCP1 may differ. Regardless, while the nature of the relationship between ERβ and UCP1 requires more scrutinous investigation, it can be definitively concluded that a critical relationship between those proteins certainly exists.

A limitation of the current study was that the normal chow and HFDs used were only controlled for total fat and calorie content, not macronutrient distribution or fiber content. However, the effect of diet was not a central aim of this study; it was simply used as a means to induce obesity in all of the groups studied. Importantly, the 16-weeks HFD feeding protocol was sufficient to increase body weight and adiposity in all groups. Another limitation is that mitochondrial respiration was only studied in PGAT and these experiments were not powered to detect sex differences due to small n’s. Future work should access these other adipose tissue depots and both sexes for the effect of ERβ on mitochondrial respiration. Another consideration in interpreting the results of this study is that the acute effects of CL were not investigated here, but rather the chronic effects of daily CL treatment for 2 weeks. Future studies need to assess how ERβ may influence acute lipolytic responses. Also, unfortunately, our UCP1 staining did not work in this current study and we were unable to validate our protein data with UCP1 staining; however, we have validated this relationship in previous studies (Clookey et al., 2019). Finally, to fully understand the mechanisms by which ERβ (both classical and non-classical functions) affect adipocyte metabolism, complete knock down of ERβ needs to be performed, as well as adipocyte-specific knock down. Other areas of future work include characterization of the molecular mechanisms by which non-classical functions of ERβ can be activated by various ligands, subcellular organelle-specific effects, and the role that ERα plays in these effects.

## Conclusion

Targeting WAT mitochondrial function (e.g., induction of WAT browning) is a viable approach for treating cardiometabolic dysfunction in both sexes, yet there are not currently safe and effective therapies for selective targeting of these adipocyte-specific pathways. Activation of specific aspects of ERβ signaling may be one novel approach to doing this in a safe, non-feminizing way. This work highlights a novel role of ERβ in the WAT browning phenomenon, demonstrating for the first time *in vitro* the necessity of ERβ for adipocyte UCP1 expression and identifying ERβ as an important potential target to activate protective adipocyte mitochondrial responses involving UCP1. We confirm our previous work both *in vivo* and *in vitro* demonstrating a strong, positive, and physiologically relevant relationship between ERβ and UCP1 expression in WAT and prove that the DBD of ERβ (i.e., classical genomic ERβ signaling) is not required for UCP1 upregulation by CL. Contrarily, we show that disruption of classical genomic signaling through ERβ may sensitize cells to the UCP1-inducing effects of CL. This was not the case in our previous study, where classical genomic signaling through ERβ was shown to be required for exercise-induced UCP1 induction in the setting of ovariectomy. In summary, this research highlights the benefits that targeting WAT ERβ may have in treating metabolic disease and emphasizes the potential role that ERβ signaling plays in modulating mitochondrial function. Finally, it draws attention to the fact that ERβ has functions independent of its ability to regulate ERE-responsive genes, and that such functions may serve as new therapeutic targets to prevent and mitigate obesity and related metabolic diseases.

## Data Availability

The raw data supporting the conclusions of this article will be made available by the authors, without undue reservation.
