# Supplementary figures and images for: Suppression of estrogen receptor beta classical genomic activity enhances systemic and adipose-specific response to chronic beta-3 adrenergic receptor (β3AR) stimulation
Source: Front Physiol. 2022 Sep 16;13:920675. doi: 10.3389/fphys.2022.920675 (PMC9534559; doi:10.3389/fphys.2022.920675)

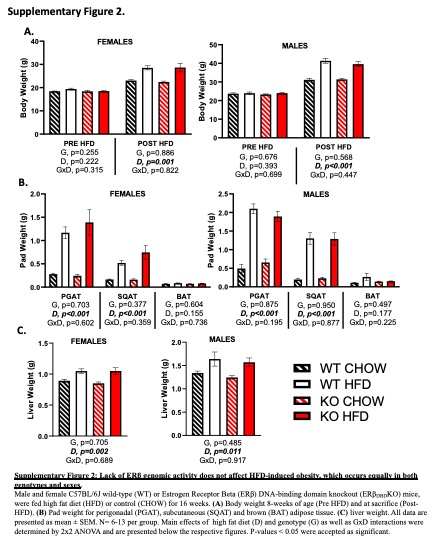

Supplement: Supplementary file 1 [file Image3.JPEG]

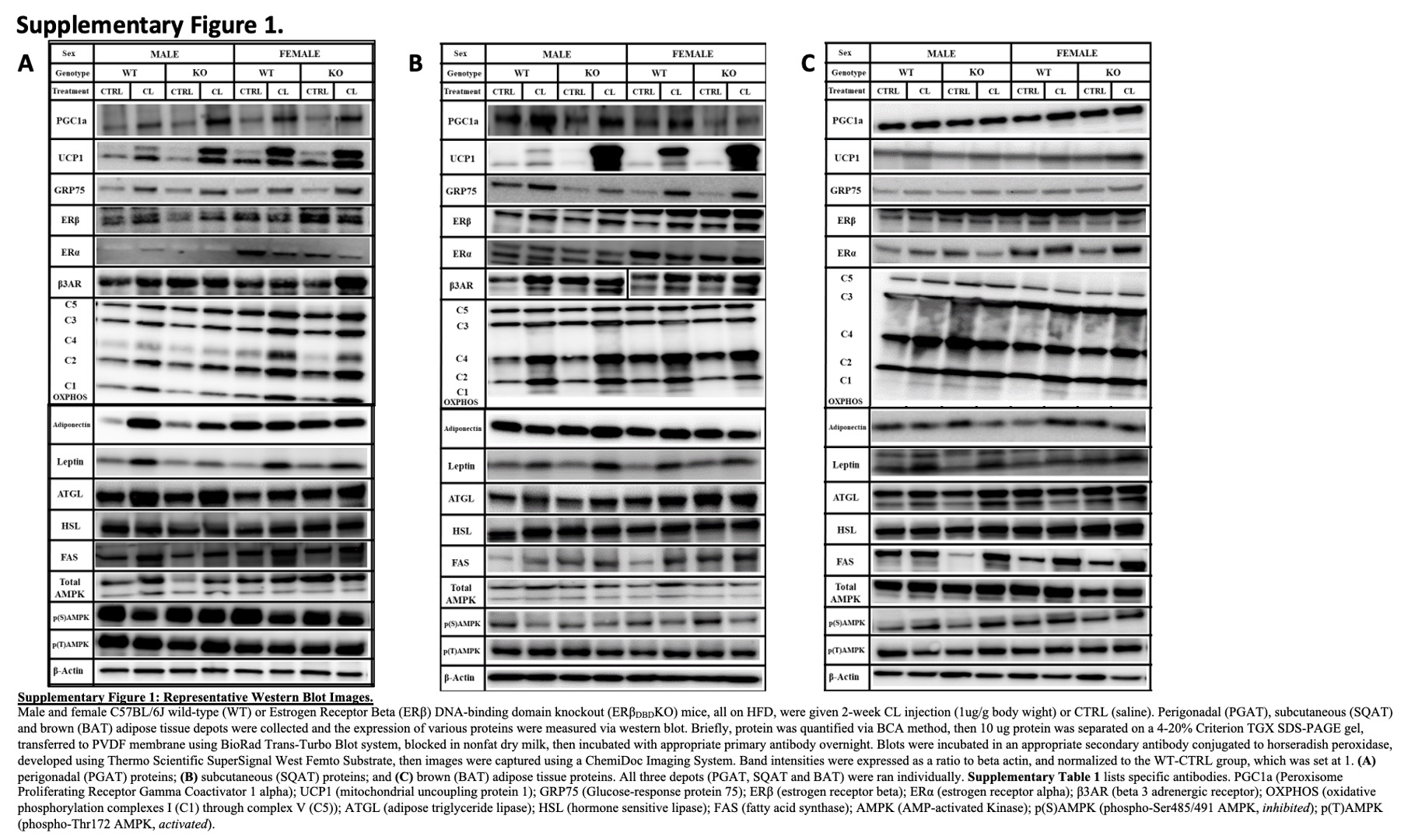

Supplement: Supplementary file 2 [file Image1.JPEG]

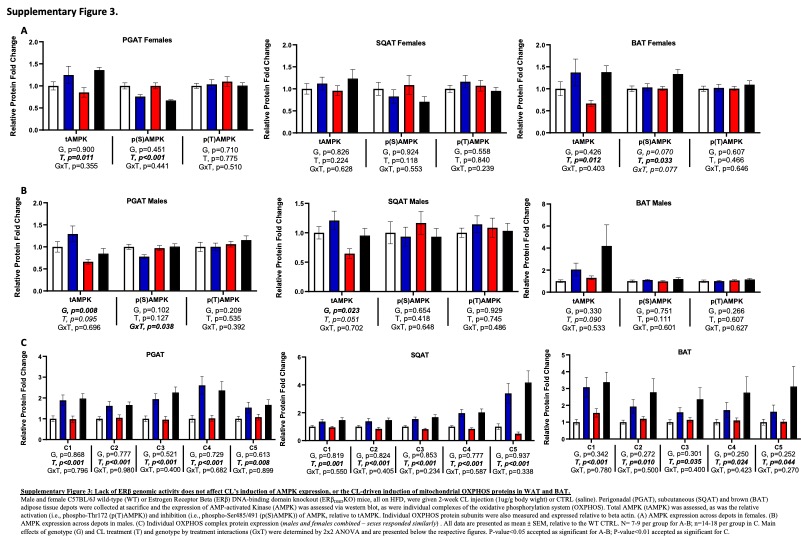

Supplement: Supplementary file 3 [file Image4.JPEG]

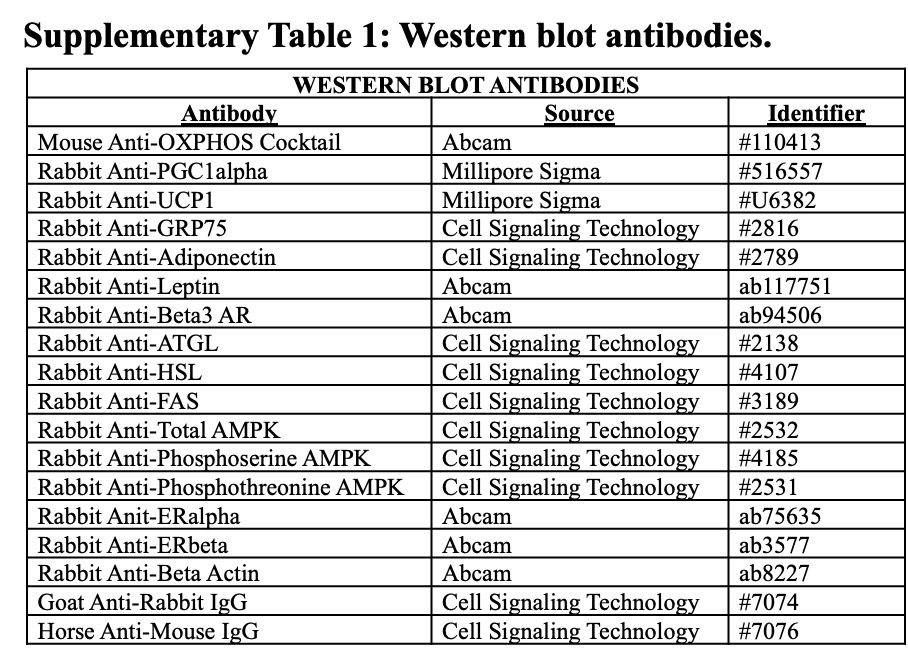

Supplement: Supplementary file 4 [file Image2.JPEG]
